# Considerations for integrating wearables into the everyday healthcare practice

**DOI:** 10.1038/s41746-023-00820-z

**Published:** 2023-04-22

**Authors:** Dylan Powell, Alan Godfrey

**Affiliations:** Department of Computer and Information Sciences, Northumbria University, Newcastle upon Tyne, UK

**Keywords:** Biomedical engineering

## Abstract

Wearable technologies are becoming ever more popular as suggested tools for use in modern medicine. Studies evidence their growing pragmatism and provision of objective data for a more informative and personalised approach to patient care. Yet many wearables are one dimensional, despite the underlying technology being common across a large array of tools. That is primarily due to the accompanying software, unmodifiable or black box-based scripts that generally lack accessibility or modification, meaning wearables may often get discarded. Use of wearables for sustainable healthcare needs careful consideration.

## Introduction

The UK life sciences is set to enter a decade of investment, innovation, and practice, coinciding with a national vision launched in 2021^1^. Investment and innovation are in demand to combat the *other* ‘silent pandemics’ i.e., common health conditions that are omnipresent and include but are not limited to dementia, high blood pressure, diabetes, and depression, all requiring different options/tools for measurement and treatment^2^. Those varied, and diverse conditions are well researched but still greatly impact the ability to age well. Correspondingly, a 10-year UN-based strategy strives to improve lives by harmonising agencies to take actions pertaining to age and ageing by drawing notable attention to e.g., data and innovation^3^. It has been written that *information is power* and when applied and discussed within the context of healthcare, information/data is an extremely valuable commodity: it is the new blood^4^. Accordingly, data capture for improved measurement and treatment is paramount within this decade of a global agenda to improve a plethora of health conditions.

The spectral themes of data and innovation would be challenging to capture within the confines of this editorial alone. Yet, one emergent trend of interest we would like to draw attention to is within the field of data and diagnostics with the methodological shift in location of where measurement(s) and treatment(s) occur. Here, the rise and improvements in digital technologies such as wearables facilitates a realisation of the paradigm, hospitals without walls^5^. Indeed, this aligns with concepts referring to independent living, whereby the person/patient can remain in their routine environment without needing to regularly visit a hospital/clinic^6^. That has two immediate benefits such as reduced burden on (i) the patient, who may have mobility and transport limitations and (ii) the clinician, by ensuring his/her/their time is optimised for those most at need of personal attention/examination. Most interestingly, by keeping the patient at home, it evokes habitual behaviours (regular habits) which can be more informative during data capture compared to ad hoc/snap-shot assessment(s) in the hospital/clinic. It is in that context wearable-based technology is shining through, enabling prolonged and remote data capture that offers more insight and personalised approaches in healthcare.

A recent perspective in *npj Digital Medicine*, highlighted what the future of wearables may be while considering the perspectives of numerous stakeholders from diverse backgrounds (e.g., clinical medicine, academia, and commercial)^7^. The conclusion and insights from that perspective reflected that advancements in wearable technology are facilitating data capture trends greater than just superficial activity monitoring. More interestingly, data capture on disease monitoring and progression is becoming feasible with previous research demonstrating the wide utility wearables can have in a variety of health conditions from Parkinson’s disease to mild traumatic brain injury, with new opportunities afforded during remote assessment^8–12^. Taking the leap of routinely using wearables (of any description) in everyday clinical life across the spectrum of the medical profession is still premature but methodologies to enable transition from research playthings to care solutions are being proposed^13–15^. Thus, the potential for wearables in healthcare is enormous but challenges remain, and there are many. For the purposes of this editorial, we draw attention to the challenge of sustainability, which is often overlooked in comparison to the technical, professional, policy and social issues. We believe that opportunities exist to drive sustainable implementation through wearables in clinical practice. Firstly, we broadly discuss how wearables could routinely enable (digital) medicine and later why sustainability in healthcare needs to be considered.

## Considerations for sustainability

The rise of wearable technologies has positively impacted the capacity and opportunity to deliver personalised care with greater medical resolution. However, any rapid development and deployment of products/devices (as well as affiliated infrastructure and services) could be viewed as both an exemplar of brilliance and error in practice. Consider the physical wearable devices themselves. There are high rates of ‘hibernating’ devices where consumers hold onto devices no longer used is hindering the recycling and circular economy of wearables and mobile devices^16,17^. Additionally, there is little consideration of the materials or carbon footprint of the hardware deployed, from the materials used, durability, energy charging capacity and efficiency^16^. Thankfully, there have been exciting sustainability developments in hardware. For example, the ability to detect blood pressure and movement using a small skin patch built from inexpensive, widely available tissue paper as well as use of graphene to create a stretchable health monitor powered by the movement of the wearer to negate a reliance on (disposable) batteries^18,19^. How those innovations evolve and become more common place to meet sustainability demands is yet to be determined but suggest exciting developments in hardware, amongst others^20^.

Where are the sustainability opportunities for software? The days of closed loop/black-box commercial systems for physiological monitoring are fading away, certainly for higher level-based software that e.g., analyses data to inform clinical decisions compared to underlying machine code (firmware) to make the hardware tick/operate. Accordingly, there is a growing trend to make analysis software fully transparent so arising digital outcomes can be used with confidence and trust. Moreover, the ability to create and develop algorithms to post process wearable data is abundant through the open creation of powerful coding packages (e.g., Python, R). Those resources are made accessibly by the abundance of online video tutorials and code sharing sites (e.g., GitHub). Therefore, it can be reasoned that the proliferation of code and arising algorithms is growing considerably faster than the underlying hardware (as the latter requires more physical resources for creation). For example, consider common wearables such as accelerometer-based devices. With that hardware, (i) derived gait outcomes (e.g., step time variability) may help examine effectiveness and digital endpoint of a pharmacological treatment in Parkinson’s disease^11^ while (ii) intensities of physical activity may be the useful outcome in managing diabetes^21^. Although seeking different outcomes to different research questions, both study examples are linked by hardware technologies (accelerometery) to detect movement (one for gait and the other for physical activity). Consequently, possible methods of sustainability emerge: the underlying sensing is of equivalence, but the software determines how the accelerometery data is interpreted. Theoretically, generic accelerometer (and/or gyroscope) based wearables could be used in a ‘circular’ fashion across the field of medicine but the software would/could be prescribed to address different clinical questions. Within the field of gait research alone there are a range of different algorithms that could be prescribed^12^ but there is a lack of ICT infrastructure and harmonisation between academic research, commercial entities, and healthcare practitioners. In short, decoupling software from the hardware may drive sustainability through flexibility.

## How might software prescriptions work?

The following examples are within the context of low-resource, observational or remote assessment and may provide some food for thought. Recently, some of the same authors highlighted wearable opportunities within the topic of mild traumatic brain injury (mTBI) arising in sports but the prevalence of mTBI is much broader when considering e.g., older adult fallers or road traffic accidents^9,22^.

Those working as first responders in the community (or in sport) often have the challenge of making rapid decisions on the severity of a head injury. Consider the scenario that a generic accelerometer-based wearable could be programmed with a validated algorithm and then attached to the person with suspected mTBI to assess their gait and balance, offering the responder more objective and high-resolution data to better inform their decision-making process. Alternatively, consider a scenario that the person may have repeated a mTBI (from playing e.g., rugby or American football or perhaps it is an older adult who is a repeated faller)^23^. Perhaps they already have the accelerometer at home from a previous meeting with the responder or it is embedded within a watch that was purchased for private use. The latter (commercial devices) may negate some of the challenges of deploying useful wearables already equipped with useful hardware (e.g., accelerometers) but providing ubiquitous sensing to healthcare. Indeed, the proliferation of consumer driven digital technologies and 5G-enabled telemedicine could enable decentralised real-time multidisciplinary decision-making involving doctors, first responders and other healthcare professionals. By relaying vital signs and live images to trauma centres, alongside the data captured from wearables, second opinions could be used to help triage and prioritise those with the most urgent clinical need for hospital intervention, while enabling those with less severe clinical need to be monitored in alternative settings during their recovery. This may help reduce the risk of unnecessary A&E assessment while improving the reassurance provided to patients during their recovery^24^.

## Why sustainability needs to be considered

Globally, healthcare is a significant contributor to net carbon emissions, which would equate to be the 5^th^ largest contributor if it were a country^25^. Therefore, attention should be focussed on implementing sustainable practice in healthcare design and future strategies. Recently, the UK-based National Health Service set two ambitious aims of (i) becoming net carbon zero through direct emissions by 2040 (e.g., on site hospital energy and water consumption) and (ii) reducing indirect emissions by 80% around 2036, which is greatly influenced by e.g., information and communication technology (ICT), and medical devices. Accordingly, there is a real and pressing demand for reliable alternatives in those current examples to aid healthcare services to improve their environmental and economic impact while striking a careful balance in maintaining robust and clinically suitable healthcare services^26,27^.

## Conclusion

Wearables should be carefully considered against the wider determinants of health, where sustainability is of utmost importance. Defined impact will only be possible when viewing change through an ecosystem which will bring about the desired actions and outcomes much faster than without collective action. Wearables could directly provide more sustainable healthcare as well as indirectly offering personalised and decentralised approaches in medicine. Fundamental sensing requirements exist across a range of conditions (silent pandemic) but actioning novel approaches through prescribed software could provide the necessary steps in the overall journey in concurrent health and sustainability ambitions.

## Wearables: opportunities

- Improved objectivity and precision in assessment offered through remote assessment,
- Increased equity and access regardless of location and or geography,
- Opportunities to innovate with analytics due to low barriers to entry with accessible software,
- Developments in product durability and longevity.

## Wearables: threats

- Emissions associated with storing of data,
- Typically, high changeover of devices by consumer, but low recycling rates and many ‘hibernating’ devices,
- Software locked to hardware.
